# Programme theory development and formative evaluation of a provincial knowledge translation unit

**DOI:** 10.1186/s12961-019-0437-y

**Published:** 2019-04-11

**Authors:** Denise Thomson, Stephanie Brooks, Megan Nuspl, Lisa Hartling

**Affiliations:** 1 Alberta Research Centre for Health Evidence, Department of Pediatrics, University of Alberta and Alberta SPOR SUPPORT UNIT Knowledge Translation Platform, 4-476 Edmonton Clinic Health Academy (ECHA), 11405 87 Ave NW, Edmonton, AB T6G 1C9 Canada; 2 Alberta SPOR SUPPORT UNIT Knowledge Translation Platform, 3-62B Heritage Medical Research Centre (HMRC), 11207 87 Ave NW, Edmonton, AB T6G 2S2 Canada; 3 Alberta SPOR SUPPORT UNIT Knowledge Translation Platform, 4-482D Edmonton Clinic Health Academy, 11405 87 Avenue, Edmonton, AB T6G 1C9 Canada

**Keywords:** Knowledge translation, Patient-oriented research, Evidence-based decision-making, Implementation science, Health research capacity-building, Communities of practice, Health research funding agencies, Evidence-informed practice, Needs assessment, Formative evaluation

## Abstract

**Background:**

Research shows a significant gap between healthcare research and evidence-based healthcare policy and practice. Knowledge translation (KT) has an important role in addressing this gap by bolstering evidence-informed healthcare. Canada’s Strategy for Patient-Oriented Research (SPOR) is a nationally mandated and supported initiative developed to respond to the gap between research and practice. One aspect of SPOR is the provincial/territorial SUpport for People and Patient-Oriented Research and Trials (SUPPORT) Units, intended to assist local health researchers and systems to reach the goal of improving the quality and quantity of patient-oriented research in Canada. This article presents the programme theory development and a formative evaluation of the KT Platform in Alberta’s SPOR SUPPORT Unit.

**Methods:**

We used a mixed-methods approach to develop the KT Platform’s programme theory and subsequently conducted the formative evaluation. An extensive needs assessment, comprised of 59 qualitative interviews with researchers and health systems employees in Canada with an interest in KT, served as the basis for our programme theory design. Three years after launching the KT Platform, we hired an evaluation consultant to conduct a formative evaluation of the Platform’s programme theory and operations. The evaluation was performed by conducting nine interviews with KT Platform service users (*n* = 6) and KT experts acting in advisory capacities to the KT Platform (*n* = 3).

**Results:**

The KT Platform developed a ‘4C Model’ as a summary of the Platform’s programme theory. This model is designed to meet local needs for capacity-building, a community of practice, consultation services, and contributions to KT science. This suite of services was found to help the local health system implement health evidence with measurable positive health outcomes. However, the community remains hesitant about their capacity as individuals to design and perform important KT activities independently.

**Conclusions:**

With the mandate and support provided by SPOR, the KT Platform was able to design a strong programme theory based on evidence from an extensive needs assessment of the local community. The resulting 4C Model has provided a framework for KT work to assist in improving local health outcomes and can be considered by others designing KT programmes as a useful model to follow. Ongoing monitoring and assessment are required to continue to identify and respond to local needs.

## Background

Research shows a significant gap between healthcare research and evidence-based healthcare policy and practice [1, 2]. Slow uptake of evidence into practice has been connected to scarce training opportunities, lack of policy support and insufficient dissemination infrastructure [3, 4]. Health systems, funders and research communities increasingly place value and efforts on implementation science – the effective synthesis and implementation of evidence – to bridge the gap between evidence-based interventions and their uptake in clinical practice [3–7]. In order to increase evidence-based healthcare decisions and protocols, including improving practices and knowledge of implementation strategies, the Canadian Institutes of Health Research (CIHR) established the Strategy for Patient-Oriented Research (SPOR) in 2013 [8].

Patient-oriented research is defined as a “*continuum of research that engages patients as partners, focuses on patient-identified priorities and improves patient outcomes. This research, conducted by multidisciplinary teams in partnership with relevant stakeholders, aims to apply the knowledge generated to improve healthcare systems and practices*” [8]. SPOR is a nation-wide initiative with various forms of support existing at the federal, provincial and territorial levels. The healthcare system in Canada is governed at the provincial and territorial level. Thus, SPOR initiated provincial and territorial multidisciplinary platforms called SPOR SUPPORT (Support for People and Patient-Oriented Research and Trials) Units to help implement principles of patient-oriented research in order to improve health outcomes [8]. SUPPORT Units provide expertise to pursue patient-oriented research that meets local health needs and priorities while building capacity following the broader SPOR vision. SUPPORT Units incorporate staff and university faculty with expertise in patient engagement, data, methods, pragmatic clinical trials, research services, career development and knowledge translation (KT). Together, the SUPPORT Units work towards the overarching goal of increasing the quantity and quality of patient-oriented research in Canada. This article describes the efforts of the province of Alberta’s SUPPORT Unit’s KT Platform to identify and meet provincial needs.

KT is defined by CIHR as “*a dynamic and iterative process that includes the synthesis, dissemination, exchange and ethically sound application of knowledge to improve health, provide more effective health services and products, and strengthen the health care system*” [9]. The KT Platform directly aligns its operations with this definition [10], basing its research and services on patient-oriented research needs for knowledge synthesis, knowledge mobilisation, and knowledge implementation. In health research, knowledge synthesis is the process of systematically collecting and analysing existing health literature to identify best practices in health policies/procedures. Knowledge mobilisation is the process of communicating findings from knowledge synthesis to relevant stakeholders with the goal of informing meaningful change in policy or practice. Finally, knowledge implementation is the act of designing and implementing new protocols, policies or practices based on best evidence identified in the knowledge synthesis. The breadth of activities involved with KT is coupled with an equally broad field of potential practice and capacity requirements depending on which element of KT is being performed. However, for ease of reading, the remainder of this article will use the term KT to encompass all three elements.

To optimise operations, the KT Platform worked to identify stakeholder KT needs during the establishment of the province of Alberta’s SUPPORT Unit. Subsequently, the KT Platform developed and implemented a programme to address those needs. This article presents (1) the development of the Alberta KT Platform programme theory in light of a needs assessment of the local community with an interest in KT and (2) a formative evaluation of the services provided by the KT Platform in the first 4 years of operation. Programme theory is the product of “*making explicit the underlying assumptions about how programs are expected to work*” [11], which can then be used to guide the evaluation of programme function and impact. The results are discussed in relation to existing literature to present opportunities and ongoing challenges faced by a KT Platform that has dedicated funding and support in place.

## Methods

We employed a mixed-methods approach to develop and monitor the KT Platform’s contribution to Alberta’s KT community as guided by the needs of key stakeholders [12]. First, we conducted a needs assessment through expert consultation to develop and define the KT Platform’s programme theory. Second, we conducted a formative evaluation of the KT Platform’s ability to follow the programme theory and perform important KT activities required to achieve the goals of patient-oriented research. The needs assessment was comprised of interviews with 43 researchers and government practitioners in Alberta with an interest in KT. These data were supplemented by interviews with another 16 experts across Canada, including individuals running other KT platforms or organisations (Table 1). Interviews were held between October 2014 and February 2016. Experts were identified through snowball sampling starting with the KT Platform’s stakeholder groups. The KT Platform local stakeholder groups include (but are not limited to) other Alberta SUPPORT Unit platforms, academic institutions (University of Alberta, University of Lethbridge and University of Calgary), the provincial health system (Alberta Health, Alberta Health Services), funders (Alberta Innovates Health Solutions), units within our health service provider organisation (Alberta Health Services) that facilitate research and improved quality of care (Strategic Clinical Networks), KT researchers and practitioners, and local health organisations [13].

**Table 1:** Experts consulted

Location of experts (*n*)	Affiliation with knowledge translation (KT) (*n*)
British Columbia (3)	KT Researcher (3)
Alberta (43)	Government (17)
	KT Researcher (26)
Manitoba (1)	KT Platform (1)
Ontario (6)	Government (1)
	KT Platform (2)
	KT Researcher (3)
Maritimes (4)	KT Platform (2)
	KT Researcher (2)
Territories (2)	Government (1)
	KT Platform (1)
Total = 59	Government (19)
	KT Platform (6)
	KT Researcher (34)

Using an open interview structure, we asked interviewees to share perspectives on their current KT capacity and practices and ways to move forward, focusing on what supports were required to increase current KT efforts in Alberta. Perspectives were captured with detailed written notes and comprised the basis of the KT Platform programme theory development. The programme theory was designed to align with the current needs of the Albertan KT community (presented in the Results section).

From May to July 2017 (3 years after beginning KT Platform operations), an external evaluation consultant conducted a formative evaluation by interviewing a sample of programme users to assess the extent to which the KT Platform was progressing according to the programme theory [12]. Nine qualitative interviews were conducted, all of 30 to 60 min in length. Interviewees included KT experts acting in advisory capacities to the KT Platform (*n* = 3) and clients of the KT Platform who had received KT Platform services beyond simple advice (*n* = 6). Perspectives collected from both the theory development and the formative evaluation took place in one-on-one interviews either in-person or over the telephone.

Data collected from interviews were entered into NVivo 11 qualitative data management software and coded inductively to conduct thematic analyses [14]. The needs assessment and formative evaluation of activities fall under article 2.5 ‘Activities Not Requiring REB Review’ in Canadian Institutes of Health Research, Natural Sciences and Engineering Research Council of Canada, and Social Sciences and Humanities Research Council of Canada, Tri-Council Policy Statement: Ethical Conduct for Research Involving Humans, December 2010, which states “*Quality assurance and quality improvement studies, program evaluation activities, and performance reviews, or testing within normal educational requirements when exclusively for assessment, management or improvement purposes, do not constitute research for the purposes of this Policy, and do not fall within the scope of REB review*”. Therefore, a formal ethics review was not required. However, before commencing each interview, interviewees were informed that their participation was completely voluntary and that their identities would remain anonymous.

## Results

### Needs assessment and programme theory development

**Fig. 1:**
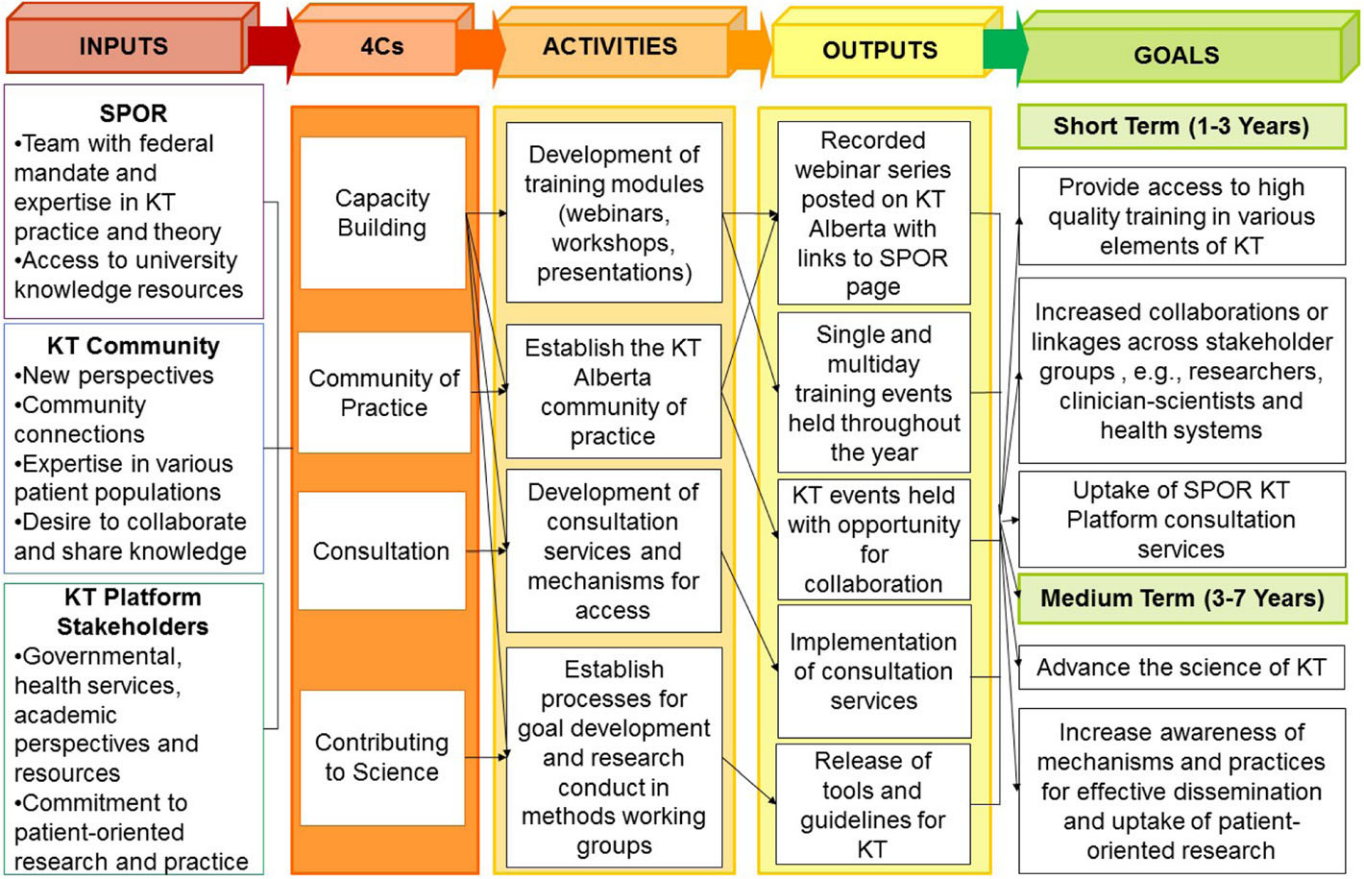
KT Platform 4C programme theory. *SPOR* strategy for patient-oriented research, *KT* knowledge translation, *4Cs* capacity-building, community of practice, consultation, contributing to science

From the needs assessment, interviewees identified four priority areas of need in Alberta, namely (1) capacity-building for trainees, practitioners and non-academic personnel in industry and government; (2) more opportunities to collaborate and share knowledge; (3) better science for evidence-based KT planning; and (4) development of consultation services to guide effective KT. These needs overlap in various ways. For example, the need for capacity-building is present in all areas. However, there are also distinct characteristics in each area. To ensure that both overarching and distinct needs were met, the KT Platform created what we called a ‘4C Model’ to guide its operations (Fig. 1). The 4C Model was developed to meet local needs for capacity-building, a community of practice, consultation services, and contributions to KT science. Rather than operationalising any existing models of large-scale KT initiatives, the 4C Model is the product of a community-based approach that meets the needs of the local context, which the KT Platform was designed to serve.

#### Capacity-building

Regardless of profession or location, interviewees stated concern about availability and accessibility of training and capacity-building. They acknowledged that opportunities to conduct KT activities exist across fields and at different levels of research and governance. In turn, interviewees emphasised the need for training opportunities, both within and outside of academia, for students, early career researchers, health professionals, administrators, government employees and people working in industry.

##### KT Platform response

In efforts to maximise accessibility and reach of KT training modules, the KT Platform decided to deliver training in various levels of formality and duration using varied formats, including webinars, workshops, lunch and learn sessions, conferences, and other training events. For example, the KT Platform developed and released a series of webinars and workshops to support the development of KT practitioner skills [15]. Additionally, the KT Platform partnered with other organisations, such as KT Canada and Cochrane, to develop and deliver training content on advanced KT topics and knowledge synthesis methods through workshop series.

#### Community of practice

Capacity-building through collaboration was emphasised in that there is no central place or established mechanism to connect, share knowledge, learn from one another, and build stronger KT teams/efforts through collective action. Specifically, interviewees emphasised that, to conduct effective KT activities, they would need to improve or cultivate collaboration with other SPOR platforms, between researchers at different institutions, with end users and patients, and with health delivery services. At the time of the interviews, participants did not feel connected enough with others in the province to establish these important partnerships.

##### KT Platform response

To help initiate and foster capacity-building and collaboration opportunities for those interested in KT and implementation science in Alberta, the KT Platform developed an online community of practice – KT Alberta [16]. KT Alberta is an inclusive, interdisciplinary community including researchers, practitioners, administrators, policy-makers and patients. The community of practice connects practitioners and organises training and networking events to facilitate knowledge sharing of KT experience in Alberta. In this way, KT Alberta efforts aim to address the time and challenges of developing important collaborations required to conduct high-quality KT and implementation science.

#### Consultation

Given the lack of training opportunities, the community called for services to provide expert input into designing, evaluating and implementing KT activities. The requirement for KT services came in two forms. First, related to the above discussion on collaboration, potential service users stated a need for consultation on who to contact as potential research users or partners and how to involve these groups. Second, again related to capacity, interviewees discussed a need for direct consultation or assistance with KT research and implementation. Common topics for consultation and direct research assistance included articulating KT plans in grant writing, evaluating KT interventions, choosing knowledge synthesis methods, and conducting KT planning and implementation based on best practices.

##### KT Platform response

The direction from Alberta Innovates and CIHR for the provincial/territorial SUPPORT Units mandated the provision of support services, as described in the Background section [8, 10]. The KT Platform’s services acknowledged provincial needs by developing two streams of consultation services, namely (1) a knowledge synthesis stream staffed to support a variety of review products with diverse purposes (e.g. support grant applications, practice guidelines and clinical pathways), and (2) a knowledge mobilisation and implementation stream designed to assist with both integrated and end-of-grant KT planning and grant writing, provide information on best practices for KT, advise on stakeholder engagement and help evaluate KT interventions [10]. Consultation is especially important for demanding academic tasks such as knowledge synthesis, evidence appraisal and programme evaluation design. Depending on the needs of a given client, consultation activities operate on a spectrum from simple advising to embedding a KT Platform staff member into a health science/service team for a period of time. At the latter end of the spectrum, an embedded KT expert can help to build capacity by providing on-site experiential training to team members or they may conduct a piece of work as appropriate. For example, a KT Platform statistician regularly performs meta-analysis for teams that would not need a statistician more frequently.

#### Contribution to science

In discussing needs for capacity-building, collaboration opportunities and consultation services, interviewees commonly mentioned the lack of resources or KT scientists in the province that could provide information on best practices in KT, especially on KT theories. Specifically, interviewees stated needs for access to more evidence/guidance on how to efficiently synthesise research findings, and confidently design, evaluate and implement KT plans.

##### KT Platform response

The KT Platform has funding dedicated to KT methods development, and thus has established a number of methods working groups to create knowledge around the various elements of KT. The methods working groups are a KT Platform initiative guided by participatory principles in which topics of KT research are identified, through discussion and consensus, by community members or a formally established advisory board. In addition, working group membership is varied to involve platform staff, academics, trainees and health service employees province-wide with an interest in implementation science. Methods working groups meet every 2 to 3 weeks (on average) over the course of each project to collectively conduct research from topic conceptualisation through manuscript development. One example of a working group project was the development of a practical guide for use of different KT models and frameworks to collect, interpret and report evidence [17]. Current working groups are examining different strategies for knowledge mobilisation (e.g. narrative, social media) and methods to increase efficiencies in knowledge synthesis. As the consultancy services expand, the KT Platform will be in a unique position with the ability to test and monitor KT activities from real-world KT efforts.

### Formative evaluation

In terms of addressing needs in the KT field, the formative evaluation showed that the KT Platform consultation services were successful in assisting in KT with measurable changes in practice and improved patient outcomes across a number of Alberta Health unit sites. For example, one of the participants represented a project the KT Platform supported with an embedded KT expert to co-create a strategy to implement a new therapy (basal bolus insulin therapy) to improve in-hospital management of individuals with diabetes. Since implementation, this programme produced a 13% absolute increase in ordering of the therapy with a significant 2% reduction of hyperglycemic patient-days resulting in a 14% decrease (2.4 days) in length of hospital stay [18]. The participants reported KT Platform support as an important part of this success, particularly around identifying barriers and enablers and possible strategies to address them.

In contrast, however, other findings from the formative evaluation underscored the ongoing need for capacity-building and training opportunities in the province. While yielding desired results in terms of patient outcomes, consultation in itself did not equal KT capacity-building in the KT Platform clients interviewed for the evaluation. This was especially true for those seeking assistance with knowledge synthesis requirements such as building and conducting literature searches and analysing collected literature. Furthermore, clients did not feel they could independently apply KT frameworks to support the implementation of research findings on the ground.

The interviewees in the formative evaluation had more concrete experience with the KT Platform than those in the needs assessment. When asked about future directions given the KT Platform’s services and goals, the participants had very specific areas of interest based on their profession. Those from academia tended to focus on the continuation of project-specific consultation services, grant-writing support and training development for students. In contrast, KT practitioners were interested in implementation science, specific collaborations to build and maintain, and training for non-academics including clinician-researchers working in health settings. Finally, those with policy perspectives focused on systems-level issues in KT such as scalability and generalisability of KT interventions.

## Discussion

To successfully conduct KT research and implementation activities, practitioners need to understand the theories and models that guide KT design, be able to rigorously synthesise relevant literature, have the qualitative skills to analyse contexts that might act as facilitators or barriers to evidence uptake, and be able to evaluate KT practices [4, 19]. KT training programmes and repositories designed to improve capacity have emerged in Canada, including the KT Canada Summer Institute [19], the Scientist Knowledge Translation Training [20], and the National Collaborating Centre for Methods and Tools [21]. However, remembering that KT encompasses knowledge synthesis, mobilisation and implementation, the breadth of required capacity complicates any one initiative’s ability to offer comprehensive capacity-building programmes. For example, those working in knowledge synthesis need knowledge and confidence in information science and database searching. Their work also often requires skills in statistics to bring together results from multiple complex studies in meaningful ways. Conversely, individuals working in knowledge mobilisation benefit from understandings of social science. They also require experience using various media to formulate evidence-based messages that reach different stakeholders in ways that are understandable and meaningful. Finally, those in knowledge implementation have skills in organisational culture and management, as well as in programme design and evaluation. This is by no means a comprehensive list of capacities required to successfully perform KT activities; therefore, it is not surprising that gaps in capacity-building efforts persist [19, 22]. Researchers continue to call for further capacity-building opportunities that address barriers such as the need for core competencies versus specialisation [23], accessibility of training programmes and the need to adapt to the rapidly changing implementation science field [4, 7].

The results of our needs assessment and subsequent formative evaluation reflect the needs and barriers described in the broader KT literature. Indeed, Alberta’s neighbouring province, British Columbia, conducted a needs assessment for KT in the health system broadly [24] and for capacity-building specifically [7]. They found that their population also requires funding and support in advancing KT science, building capacity in KT, managing KT projects, funding KT activities, and advocating for KT. A broader report compiling KT efforts from 15 countries worldwide to identify strategies to strengthen KT also found that lack of sustainable funding impacts the success of KT efforts [25]. Along with Australia, the United States and other countries in Europe, Asia and South America, Canada was highlighted in this study for its advanced KT landscape. This study found that “*to be fully effective, policies and programs to encourage increased research translation need to be part of a stable national innovation strategy and administered by an independent agency*” ([25], p. 15), similar to the SPOR programme in Canada. With national and provincial funding for the SPOR programme, the KT Platform is uniquely positioned to test how funding and mandate, as repeatedly called for in the KT literature, impact a community’s ability to increase KT to support patient-oriented research.

Through SPOR, the KT Platform has been able to build a programme theory based on local needs and begin to roll out relevant programme activities and services. However, our formative evaluation shows that our client base requires ongoing support before users will feel confident undertaking KT activities independently. One of the challenges related to this, and the gaps identified above, is that KT as defined in Canada has a broad scope with a vast number of activities and responsibilities in its remit. KT activities include knowledge synthesis, dissemination, translation, implementation, evaluation and ongoing monitoring, as well as stakeholder (including patient) engagement. The scope of elements involved in KT suggests that multi-disciplinary team-based approaches to KT are required, as well as varied skill sets, expertise and mixed methods.

The need for such an approach became especially apparent in the formative evaluation as the interviewees, based on their professions, stated a range of goals they felt most pertinent to future KT Platform programme refinement. Consistent with other KT needs assessments [7, 23], the divisions of interest range in (1) scope, from project-specific to policy level; (2) focus, including collaboration, planning, dissemination, application, training and science; and (3) degree of technicality requiring beginner to advanced KT skills and knowledge. The variety of KT activities and ongoing needs stated across the literature, and in our own results, highlights the need for the KT Platform to continue development and delivery of varied training opportunities as well as consultation services with the ability to embed experts into project teams. Furthermore, the establishment and upkeep of KT Alberta, the community of practice, will be vital to connect those with KT interests and needs to KT training opportunities, collaboration, knowledge and supports.

Communities of practice are “*groups of people who share a concern, a set of problems, or a passion about a topic, and who deepen their knowledge and expertise in this area by interacting on an ongoing basis*” ([26], p. 304). By coming together through formal and informal interactions, community members use, share and heighten their skills and knowledge of a given field or practice, in our case KT [27, 28]. In relation to patient-oriented research, communities of practice can facilitate KT by promoting and supporting active collaborations and research activities that bring people together to work on shared concerns and challenges [4, 29]. Furthermore, these communities can operate successfully across geographical borders [30, 31] and disciplines [32]. Studies also show that communities of practice with online components bolster the strength of KT activities [33]. However, the existence of a community of practice in itself does not guarantee health system changes, as recommendations or practice changes may be met with resistance at organisational or governmental levels [34]. Nevertheless, such communities have been shown to be successful if localised as they are increasingly relevant and dynamic in relation to changing local contexts [35]. Moving forward, the KT Platform is working to further establish and strengthen KT Alberta.

In line with existing KT literature, our interviewees in the needs assessment as well as the formative evaluation called for theoretical developments in KT in order to develop strategies and better predict outcomes of KT practices on the ground [36, 37]. KT activities are performed throughout the process of translating health research from clinical studies to practice, often with the intent to change practices, requiring behaviour changes. Studies show that behavioural research often does not account for real world barriers and facilitators [38], and thus is not considered useful by stakeholders [39]. In turn, such research can have little uptake and impact on health outcomes [40]. To develop necessary theoretical underpinnings of KT activities, research and testing is required around (1) knowledge synthesis methods that efficiently identify best intervention strategies [36, 41]; (2) understanding causal pathways operating in given interventions and their related contexts [5, 36, 42, 43]; (3) how to effectively de-implement non-evidence-based practices [44]; and (4) evaluation and monitoring of outcomes in newly implemented policies and practices [45]. Our methods working groups are conducting studies to help build understanding of KT operations such as generalisability and validity of implementation strategies [41]. They are also working towards contributing to frameworks and theories in the context of various implementation settings. Furthermore, our consultation services meet ongoing needs of ‘design for dissemination’, the practice of designing research, interventions and programmes in ways that consider how, where and by whom they will eventually be used [3, 46]. As we initiate methods working group studies connected to our consultation services, we hope to offer new understandings to implementation science from real-world settings and continue to be responsive to the needs and questions presented by our local stakeholder groups.

## Strengths and limitations

### Strengths

The KT Platform operations resulted from a well-structured programme theory driven by an extensive needs assessment. Our multi-faceted approach allowed us to respond to widespread needs, capacity-building, for example, from different angles, while also developing responses to more distinct and specific needs such as furthering implementation science on topics specific to Albertan needs. The results from our needs assessment match those commonly reported in KT literature. The positive outcomes associated with our programme activities to address these common KT needs suggest that our programme may be a model for other jurisdictions to consider when identifying and addressing local KT needs. Of course, KT approaches and activities have to be adapted to local context and needs, but our programme development methods and programme model are worth considering by others in KT programme planning stages.

### Limitations

While our needs assessment was extensive and the findings were in accordance with the KT literature and other Canadian needs assessments, our list of KT experts was not necessarily comprehensive as terminology in KT varies to the point that it is often unclear with regards to (1) who may be conducting KT activities regularly and (2) whether a given individual would identify as a KT practitioner or researcher [47]. Similarly, the small sample of participants in the formative review limits the generalisability of our results. To ensure we continue to meet our stakeholders’ needs as they arise, we have developed a system of ongoing evaluations and programme refinements.

## Conclusion

The current data and the existing literature suggest considerable room for improvement in designing for dissemination and uptake. Implementation science requires work on terminology, identifying causal pathways, and developing knowledge curation protocols that are rigorous and sharable to the wider KT community [42]. In a Western Canadian context, studies show the important role that funding agencies have in supporting and advancing KT to strengthen healthcare systems [7, 24, 48]. The results from our needs assessment, programme theory development and formative evaluation begin to highlight impacts that have emerged in early years of SPOR funding and mandate to increase the conduct and uptake of patient-oriented research. With input from the community, the KT Platform built a programme based on four core areas for KT and implementation science, namely capacity-building, community of practice, contribution to science and consultation. With SPOR funding, the KT Platform in Alberta was able to develop and offer various training opportunities and one-on-one consultation services to Albertans interested in KT. The KT Platform was also able to start building a programme of implementation science research to support local need for evidence-based KT guidance. The formative evaluation showed that our approach is moving the needle to help meet the needs of the KT community and the goals of SPOR. Continued efforts to further develop KT Alberta will help strengthen this work by acting as a knowledge and training module repository and facilitating collaboration and community across KT stakeholder groups in the province.

## Abbreviations

CIHR: Canadian Institutes of Health Research
KT: Knowledge translation
SPOR: Strategy for Patient-Oriented Research
SUPPORT: Support for People and Patient-Oriented Research and Trials
